# Clinical sensitivity and specificity of a high-throughput microfluidic nano-immunoassay combined with capillary blood microsampling for the identification of anti-SARS-CoV-2 Spike IgG serostatus

**DOI:** 10.1371/journal.pone.0283149

**Published:** 2023-03-23

**Authors:** Grégoire Michielin, Fatemeh Arefi, Olha Puhach, Mathilde Bellon, Pascale Sattonnet-Roche, Arnaud G. L’Huillier, Isabella Eckerle, Benjamin Meyer, Sebastian J. Maerkl

**Affiliations:** 1 Institute of Bioengineering, School of Engineering, École Polytechnique Fédérale de Lausanne, Lausanne, Switzerland; 2 Department of Microbiology and Molecular Medicine, Faculty of Medicine, University of Geneva, Geneva, Switzerland; 3 Laboratory of Virology, Division of Laboratory Medicine, Geneva University Hospitals and Faculty of Medicine, Geneva, Switzerland; 4 Pediatric Infectious Diseases Unit, Department of Woman, Child and Adolescent Medicine, Geneva University Hospitals and Faculty of Medicine, Geneva, Switzerland; 5 Division of Infectious Diseases, Department of Medicine, Geneva University Hospitals and Faculty of Medicine, Geneva, Switzerland; 6 Center for Emerging Viral Diseases, Geneva University Hospitals & Faculty of Medicine, Université de Genève, Geneva, Switzerland; 7 Centre for Vaccinology, Department of Pathology and Immunology, University of Geneva, Geneva, Switzerland; Kathmandu Institute of Applied Sciences, NEPAL

## Abstract

**Objectives:**

We evaluate the diagnostic performance of dried blood microsampling combined with a high-throughput microfluidic nano-immunoassay (NIA) for the identification of anti-SARS-CoV-2 Spike IgG seropositivity.

**Methods:**

We conducted a serological study among 192 individuals with documented prior SARS-CoV-2 infection and 44 SARS-CoV-2 negative individuals. Participants with prior SARS-CoV-2 infection had a long interval of 11 months since their qRT-PCR positive test. Serum was obtained after venipuncture and tested with an automated electrochemiluminescence anti-SARS-CoV-2 S total Ig reference assay, a commercial ELISA anti-S1 IgG assay, and the index test NIA. In addition, 109 participants from the positive cohort and 44 participants from the negative cohort participated in capillary blood collection using three microsampling devices: Mitra, repurposed glucose test strips, and HemaXis. Samples were dried, shipped by regular mail, extracted, and measured with NIA.

**Results:**

Using serum samples, we achieve a clinical sensitivity of 98·33% and specificity of 97·62% on NIA, affirming the high performance of NIA in participants 11 months post infection. Combining microsampling with NIA, we obtain a clinical sensitivity of 95·05% using Mitra, 61·11% using glucose test strips, 83·16% using HemaXis, and 91·49% for HemaXis after automated extraction, without any drop in specificity.

**Discussion:**

High sensitivity and specificity was demonstrated when testing micro-volume capillary dried blood samples using NIA, which is expected to facilitate its use in large-scale studies using home-based sampling or samples collected in the field.

## Introduction

After its appearance in 2019, severe acute respiratory syndrome coronavirus 2 (SARS-CoV-2) rapidly caused a global pandemic of coronavirus disease of 2019 (COVID-19), totaling over 500 million declared cases as of spring 2022 [1]. Serosurveillance was quickly recognized as an important tool to better understand the evolution of the pandemic and inform public health decisions [2]. The first published results for SARS- CoV-2 seroprevalence studies were from Stringhini *et al*. describing the seroprevalence after the first wave of the pandemic in the population of Geneva [3]. Also, a coordinated nationwide serosurveillance program called Corona Immunitas helps gather critical data on the SARS-CoV-2 epidemiological situation in Switzerland [4]. Similar serological surveillance programs were conducted in France [5], Netherlands [6], India [7], and the United States of America [8], among others [9].

In order to facilitate such serological surveys, and in particular when proposing longitudinal or repeat testing, it is critical to reduce the burden on study participants as much as possible by simplifying blood sampling and avoiding unnecessary visits to testing centers, while less invasive sampling is especially important when including children into such surveys. However, many serological surveys still rely on a visit to a healthcare center or testing facility where venous blood draws are obtained. Visits to central care facilities could present the participants and staff with a risk of infection. Moreover, some populations are difficult to include during an epidemic or pandemic when such serosurveys are of utmost importance, due to their limited access to proper facilities or due to possible vulnerabilities, such as persons living in elderly care facilities. When large-scale serosurveillance programs are conducted, costs associated with sampling, sample preparation, samples shipping, and analysis also become important parameters.

Capillary blood microsampling offers the possibility to decentralize blood collection by untrained individuals, and there is evidence of successful use of capillary blood samples in SARS-CoV-2 serological surveys. Indeed, self-collected capillary blood samples can be sent directly in liquid form using microtubes [10] or specific collection devices such as Tasso-SST [11, 12], however the collected volumes of around 250–500 μL are higher compared to microsampling, which is generally under 50 μL of blood collected. Also, facilitated logistics by shipping at room temperature with long-term stability and reduced biohazard can be achieved after drying of blood samples, while still allowing appropriate detection of anti-SARS-CoV-2 antibodies. For example, Beyerl *et al*. used at-home blood spot collection on standard filter paper cards in a large-scale serosurvey [13]. They obtained high sensitivity and specificity when extracting three sub punches of the dried blood spots and assaying the eluate in micro sample cups using the Elecsys anti-SARS-CoV-2 N total Ig assay on the Roche system.

Microsampling devices obtaining a defined blood volume have also been used, with the potential advantage of removing the hematocrit bias associated with the processing of samples directly spotted on filter paper [14]. For instance, direct collection of capillary blood microsamples on Neoteryx Mitra devices has been shown to provide comparable results to serum samples using the Roche Elecsys assay [15] or in combination with ELISAs [8, 16]. Similarly, studies showed the potential of using other volumetric microsampling systems by using minivette and subsequent transfer on filter paper [17], or using devices which, like Mitra, also allow direct microsample collection of defined volume such as Hemapen [18], Capitainer [19] or HemaXis [20].

While the performance of dried blood sample testing on existing assays is good, sample dilution upon extraction can lead to problems of lowered sensitivity [21]. Also, the small volume of the eluate can be a limitation for reanalysis, for subsequent testing of other parameters, or for biobanking applications.

To address these limitations, we previously demonstrated the possibility to detect the presence of SARS-CoV-2 anti-spike IgG antibodies in ultralow-volume blood samples using a microfluidic nano-immunoassay (NIA) validated on 289 serum samples [22]. The platform allows for high-throughput testing of up to 1024 samples in parallel on a single device, with minimal sample or reagent consumption, leading to cost-effective serological testing, and excellent assay performance with 100% specificity and 98% sensitivity. As a proof-of-concept, we also showed the compatibility of NIA with low-volume dried blood samples by transferring EDTA whole blood on microsampling devices, followed by drying, extraction, and testing with NIA.

In this study, we aimed to quantify the performance of NIA (index test) in analyzing capillary blood samples collected on three different microsampling devices, namely 10 μL on Neoteryx Mitra, 0·6 μL on repurposed glucose test strips, and 10 μL on DBS Systems HemaXis. We set out to determine whether NIA can be used to measure SARS-CoV-2 anti-Spike IgG antibody levels in dried capillary blood microsamples and to assess and compare the so obtained sensitivity and specificity with serum samples from the same individuals analyzed by Roche Elecsys anti-SARS-CoV-2 S total Ig, Euroimmun ELISA anti-S1 IgG, and NIA.

We show the ability of NIA to classify subjects with prior SARS-CoV-2 infection 11 months post infection documented by qRT-PCR (SARS-CoV-2 positive) and subjects without known prior infection (SARS-CoV-2 negative) using serum obtained by venipuncture. Next, we present the performance of the assay when used in combination with microsampling using Mitra, repurposed glucose test strips, or HemaXis, and also present automated dried blood spot (DBS-A) extraction of HemaXis. Using serum samples, NIA shows excellent performance with clinical sensitivity of 98·33% and specificity of 97·62% in samples taken 11 months post infection. Combined with microsampling, there is no change in specificity on NIA and the sensitivity remains high at 95·05% when using Mitra device, 61·11% using glucose test strips, 83·16% with HemaXis, and 91·49% for HemaXis after DBS-A extraction. We compare the results with matching serum samples measured on the Roche Elecsys anti-SARS-CoV-2 S total Ig as reference assay and show a 98·20% agreement when measuring serum samples on the microfluidic NIA index test, as well as a 95·80% agreement when combined with microsampling on Mitra, 72·55% with glucose test strips, 87·22% with HemaXis, and 93·75% after DBS-A extraction of HemaXis. Together, these results indicate a good performance of NIA when using dried capillary blood samples and validate its utility in performing serological surveys, allowing decentralized sample collection and centralized analysis with high-throughput, low sample volume requirements, and low reagent consumption.

## Materials and methods

A complete description of the materials and methods is provided as a supplement (S1 File and S1 Fig). Participants with documented PCR-positive SARS-CoV-2 infection (SARS-CoV-2 positive) and participants without knowledge of previous infection (SARS-CoV-2 negative) were invited to participate in the study and blood samples were collected by venipuncture and capillary microsampling. The samples were tested on Roche Elecsys anti-SARS-CoV-2 S immunoassay as a reference assay, a commercial ELISA Euroimmun SARS-CoV-2 anti-S IgG, and on a microfluidic nano-immunoassay [22]. For the Roche Anti-SARS-CoV-2 S reference assay, studies performed independently showed high specifictity and sensitivity of 99.95% (95% confidence interval [CI]: 99.87–99.99; 7876/7880) and 97.92% (95% CI: 95.21–99.32; 235/240), respectively, for samples ≥14 days post-PCR [23]. This study was approved by the local ethics committee (CCER project numbers 2020–00516 and 2020–02323) and registered (NCT04329546) prior to initiation. For all participants, a written informed consent was obtained.

## Results

### Participants

**Fig 1:**
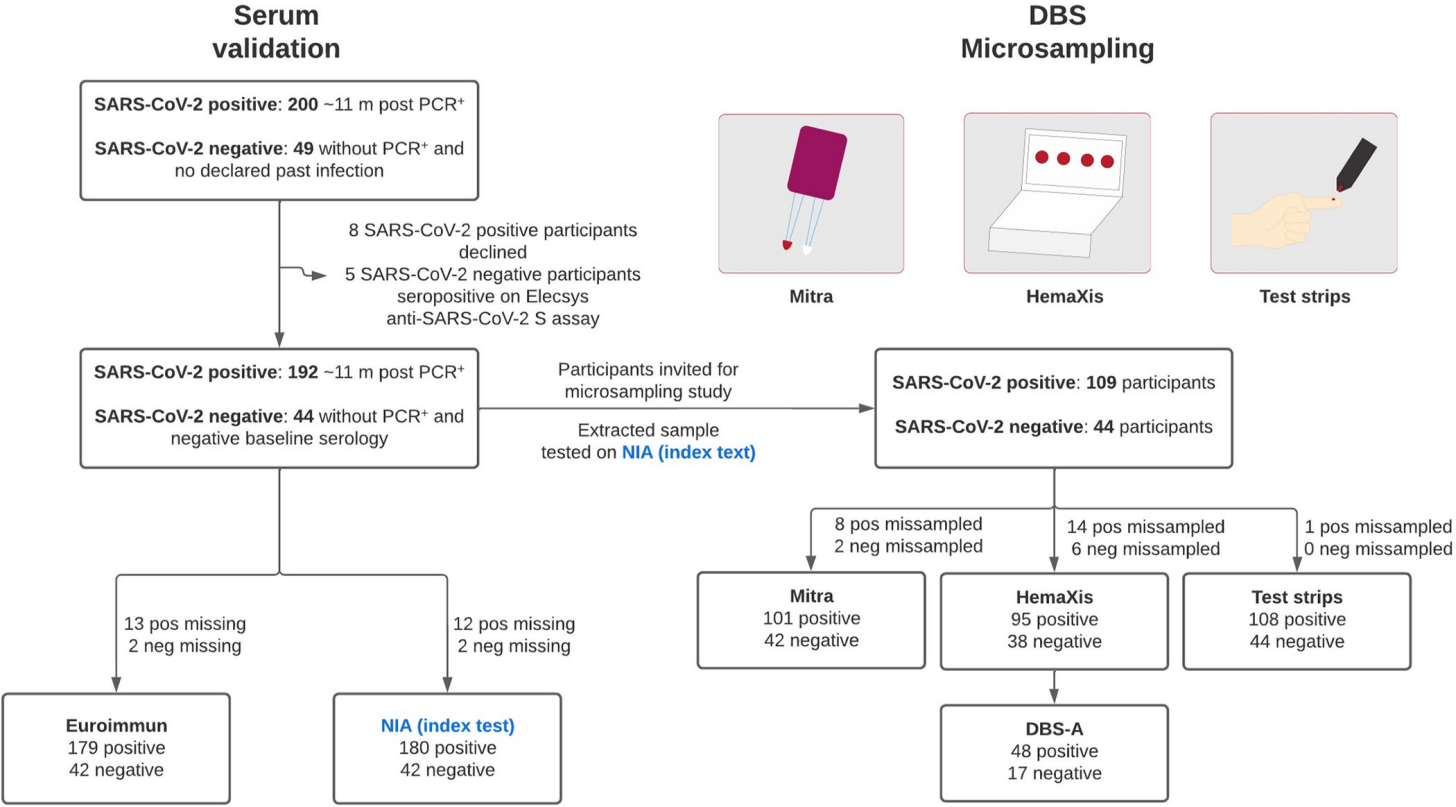
Study participants. Serum validation) 249 participants were included in this study and were invited for a venipuncture. Serum obtained was used for testing on the reference standard Roche Elecsys anti-SARS-CoV-2 S total Ig assay, Euroimmun anti-S1 IgG assay, and on the microfluidic nano-immunoassay (NIA) index test. DBS microsampling) On the same day, the participants were invited to perform a capillary blood collection using three different microsampling devices: Mitra, repurposed glucose test strips, and HemaXis. The samples were shipped by regular mail in a dried state and extracted upon reception. For filter cards collected with the HemaXis device, a subset of samples was extracted by flowthrough desorption using an automated instrument (DBS-A, Gerstel AG).

The description of the study is presented in Fig 1. We invited 200 participants enrolled in an ongoing longitudinal serological study using venous blood with documented SARS-CoV-2 infection by positive qRT-PCR [24], and 192 (96%) participated in this study consisting in a follow-up visit 11 months post infection (SARS-CoV-2 positive group). In addition to a venipuncture, 109 subjects (56·77%) agreed to take part in the microsampling study. As prepandemic capillary samples were not available for the different microsampling devices, the subjects in the SARS-CoV-2 negative group consisted of 49 individuals without known past infection who were recruited specifically for this study, although prior infection could not be excluded with certainty in these 49 individuals. Based on the high specificity of Elecsys anti-SARS-CoV-2 S assay, 5 participants in the SARS-CoV-2 negative group who tested positive were excluded from the study. All 44 participants in the SARS-CoV-2 negative group had both venipuncture and microsampling performed. Participants in the cohort had a median age of 41·21 (interquartile range: 30·98–50·98) and 174/249 (69·86%) were women.

### Blood sampling

For capillary blood microsampling, participants were provided with two commercially available microsampling devices: Neoteryx Mitra and DBS System HemaXis. We also tested repurposed glucose test strips as a low-cost, ultra-low volume alternative (Fig 1). Microsampling was performed on the same day as the venipuncture, and 153 individuals participated in micro-volume capillary blood sampling (62·70% of the total). Each participant received a kit containing all microsampling devices and three lancets to ensure the possibility of repeating a fingerprick if needed between the different collections. Participants received the suggestion to first collect capillary blood samples on the Mitra device, followed by the glucose test strips and then the HemaXis device, and we note that this order could influence sample collection success rate. Except for one participant who collected the samples at home, all samples were collected at the Geneva University Hospital with the supervision and help of research staff.

Based on visual inspection of the collection tip for presence or absence of blood, as well as appropriate sampling according to the instructions for use provided by the manufacturer, the Mitra device allowed for 143 (93·46%) successful collections on at least one of the two collection tips (2 samplers per Mitra cartridge). Among the 10 failed sampling events, 8 were excluded due to insufficient sampling, and 2 were due to oversampling manifested by blood present on the stem of the Mitra sampler. The glucose test strips allowed collection of blood in 152 (99·35%) cases, with one participant who did not collect any visible blood. We note that it was not possible to visually assess whether the 0·6 μL test strip channel was under- or over- sampled. Therefore, any glucose test strip with visible blood collection was considered as a successful collection. Sample collection with the Hemaxis device was successful in 133 (86·93%) of cases, with participants having at least one correctly sampled blood spot. 22 samples had no dried blood spot or had spots of small size due to undersampling, while oversampling was not observed. As each HemaXis device contains 4 available collection channels, 65 participants (42·48%) could collect an additional third or fourth spot which were used for automated extraction using flow-through desorption (DBS-A instrument, Gerstel AG). These results show that microsampling of capillary blood results in a high sampling success rate, even when collection on multiple different devices was required.

### Sample validation in serum

We used serum samples obtained by venipuncture for measurements on the reference Roche Elecsys anti-SARS-CoV-2 S total Ig assay, on Euroimmun ELISA anti-S1 IgG assay, and on NIA for initial validation. We tested a total of 192 samples from subjects with prior SARS-CoV-2 infection (SARS-CoV-2 positive) and 44 samples from subjects without prior infection (SARS-CoV-2 negative) with the Elecsys anti-SARS- CoV-2 S total Ig reference assay, 179 SARS-CoV-2 positive and 42 SARS-CoV-2 negative with the ELISA anti-S1 IgG assay, and 180 SARS-CoV-2 positive and 42 SARS-CoV-2 negative on the NIA assay (Fig 1). The difference in the number of samples tested is explained by serum samples being initially tested on the reference Elecsys assay, while samples were retrieved at a later timepoint only when sufficient volume was available for the measurement on ELISA and NIA assays.

**Fig 2:**
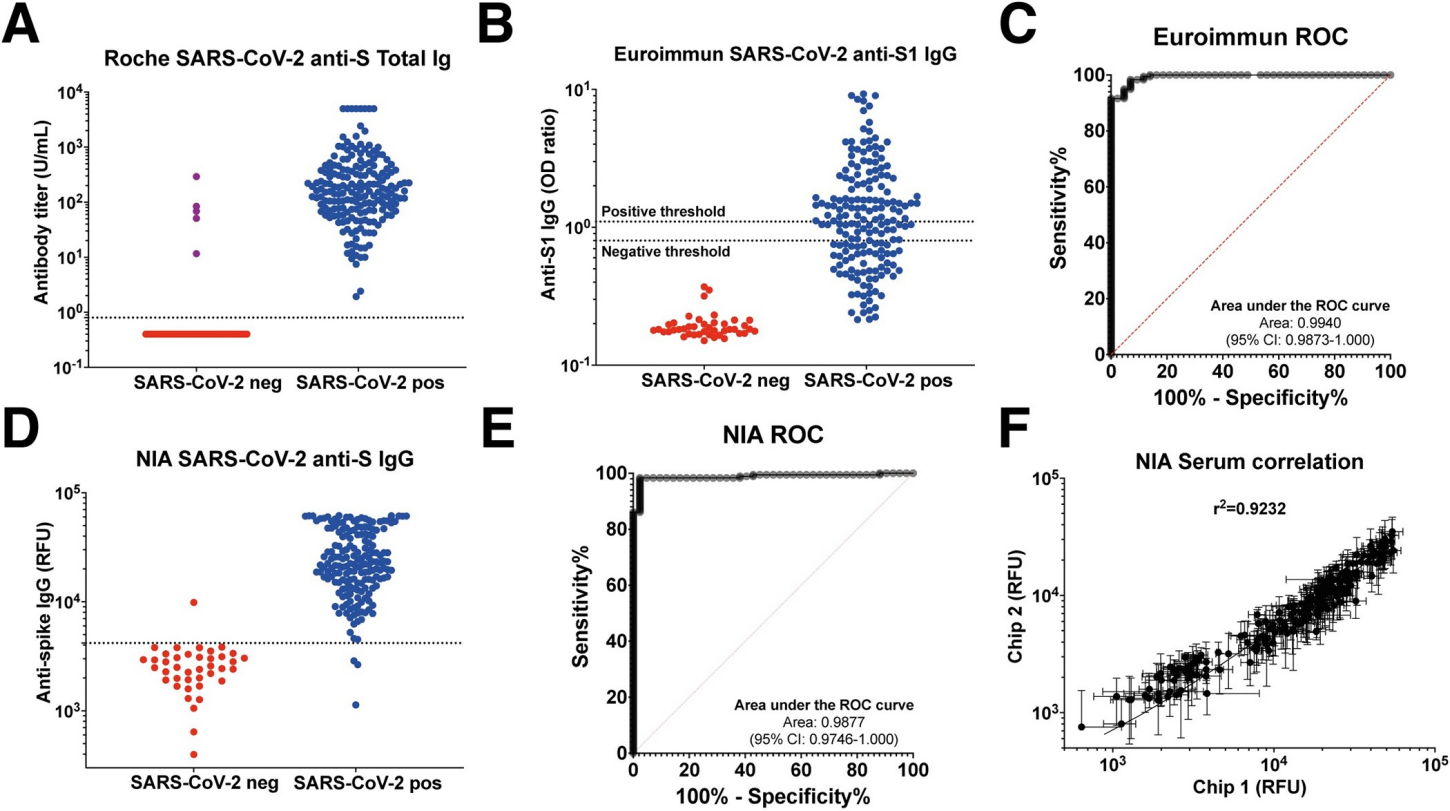
Sample validation with Roche Elecsys, Euroimmun ELISA, and NIA. A) Roche Elecsys anti-SARS- CoV-2 S total Ig assay. 5 SARS-CoV-2 negative samples (purple color) with signal above the positivity threshold (>0.8) were excluded from the study. B) Euroimmun ELISA anti-S1 IgG assay. The thresholds for positivity (>1.1) and negativity (<0.8) classification as provided by the manufacturer are indicated. C) ROC curve with calculated AUC for Euroimmun ELISA. D) NIA measuring inactivated serum samples at 1:8 dilution. E) ROC curve with calculated AUC for NIA. F) Correlation between two different NIA chips (N = 198). Samples with saturating signals (>55’000 RFU) were removed from the plot and correlation calculation.

For the Elecsys anti-SARS-CoV-2 S total Ig reference assay of serum samples (Fig 2A), all 192 participants with available sample of the SARS-CoV-2 positive cohort showed a signal above the positivity threshold (≥0·8 U/mL). Among them, 8 samples had signal >5000 U/mL and the 184 remaining samples signal had a geometric mean of 140·4 U/mL (95% CI: 116·7–169·0) with an interquartile range of 68·03–343·8 U/mL.

For the Euroimmun ELISA anti-S1 IgG assay, we observe a relatively poor discrimination between SARS-CoV-2 positive and SARS-CoV-2 negative samples (Fig 2B). Based on the positivity threshold provided by the manufacturer (≥1·1), the ROC analysis returns a clinical sensitivity of 51·40% (95% CI: 44·12–58·61) and specificity of 100% (95% CI: 91·62%-100·0), resulting in a low concordance with the Roche reference assay of 60·63% observation agreement and a Kappa coefficient of 0·287 (95% CI: 0·205–0·369). Considering an approximate prevalence of 25% at that stage in the pandemic [25], this would correspond to a positive predictive value (PPV) of 100%, a negative predictive value (NPV) of 86·06% and a diagnostic accuracy of 87·85%. Finally, the receiver operating characteristic curve (AUROC) is 0·9940 (95% CI: 0·9873–1·000) (Fig 2C).

The microfluidic NIA test showed excellent discrimination between SARS-CoV-2 positive and SARS- CoV-2 negative samples when analyzing heat-inactivated serum at a 1:8 dilution (Fig 2D). The AUROC is 0·9877 (95% CI: 0·9746–1·000) (Fig 2E) and the positivity threshold determined by the maximum likelihood (>4182 RFU) gives a clinical sensitivity of 98·33% (95% CI: 95·22–99·55) and specificity of 97·62% (95% CI: 87·68–99·80). This result is similar to the clinical sensitivity observed in our previous study [22], and demonstrates the robustness of NIA in detecting the presence of anti-SARS-CoV-2 antibodies not only in subjects with recent infection, but also following a long interval of 11 months between infection and serological testing. The percentage agreement with the Roche assay was 98·20% with a Kappa coefficient of 0·942 (95% CI: 0·886–0·998) (Table 1). With a prevalence of 25%, this would correspond to a PPV of 93·23%, a NPV of 99·43% and a diagnostic accuracy of 97·79% (Table 1). We note that only three samples in the SARS-CoV-2 positive cohort have NIA signals below the threshold, while for the SARS-CoV-2 negative cohort a single sample has a positive signal on NIA leading to the observed specificity below 100%. Finally, we evaluated the reproducibility between measurements performed on two different days on NIA (N = 198) and observed a correlation with an R2 = 0·9232 (Fig 2F).

**Table 1:** Performance characteristics of microfluidic nano-immunoassay (NIA) index test.

	Sensitivity	95% CI	Specificity	95% CI	PPV	NPV	Accuracy	Agreements	Kappa	95% CI
*Serum*	** **		** **		** **	** **		** **	** **	
**Euroimmun**	**51.40%**	44.12–58.61	**100%**	91.62–100.00	**100%**	**86.06%**	**87.85%**	**60.63%**	**0.287**	0.205–0.369
**NIA**	**98.33%**	95.22–99.55	**97.62%**	87.68–99.80	**93.23%**	**99.43%**	**97.79%**	**98.20%**	**0.942**	0.886–0.998
*Microsampling*					** **	** **	** **	** **	** **	
**Mitra**	**95.05%**	88.93–97.87	**97.62%**	87.68–99.88	**93.01%**	**98.34%**	**96.98%**	**95.80%**	**0.902**	0.825–0.978
**Test strips**	**61.11%**	51.69–69.77	**100%**	91.97–100.0	**100%**	**88.52%**	**90.28%**	**72.55%**	**0.478**	0.364–0.593
**Hemaxis**	**83.16%**	74.38–89.36	**97.37%**	86.51–99.87	**91.33%**	**94.55%**	**93.82%**	**87.22%**	**0.720**	0.600–0.840
**Hemaxis DBS-A**	**91.49%**	80.07–96.64	**100%**	81.57–100	**100%**	**97.24%**	**97.87%**	**93.75%**	**0.851**	0.711–0.991

For Euroimmun, the sensitivity and specificity values were calculated using the manufacturer’s positivity threshold. Percentage agreement with the Roche reference assay is presented as well as the corresponding Kappa coefficient. Positive and negative predictive values (PPV and NPV) and accuracy are provided for a prevalence of 25%.

### Mitra microsampling

**Fig 3:**
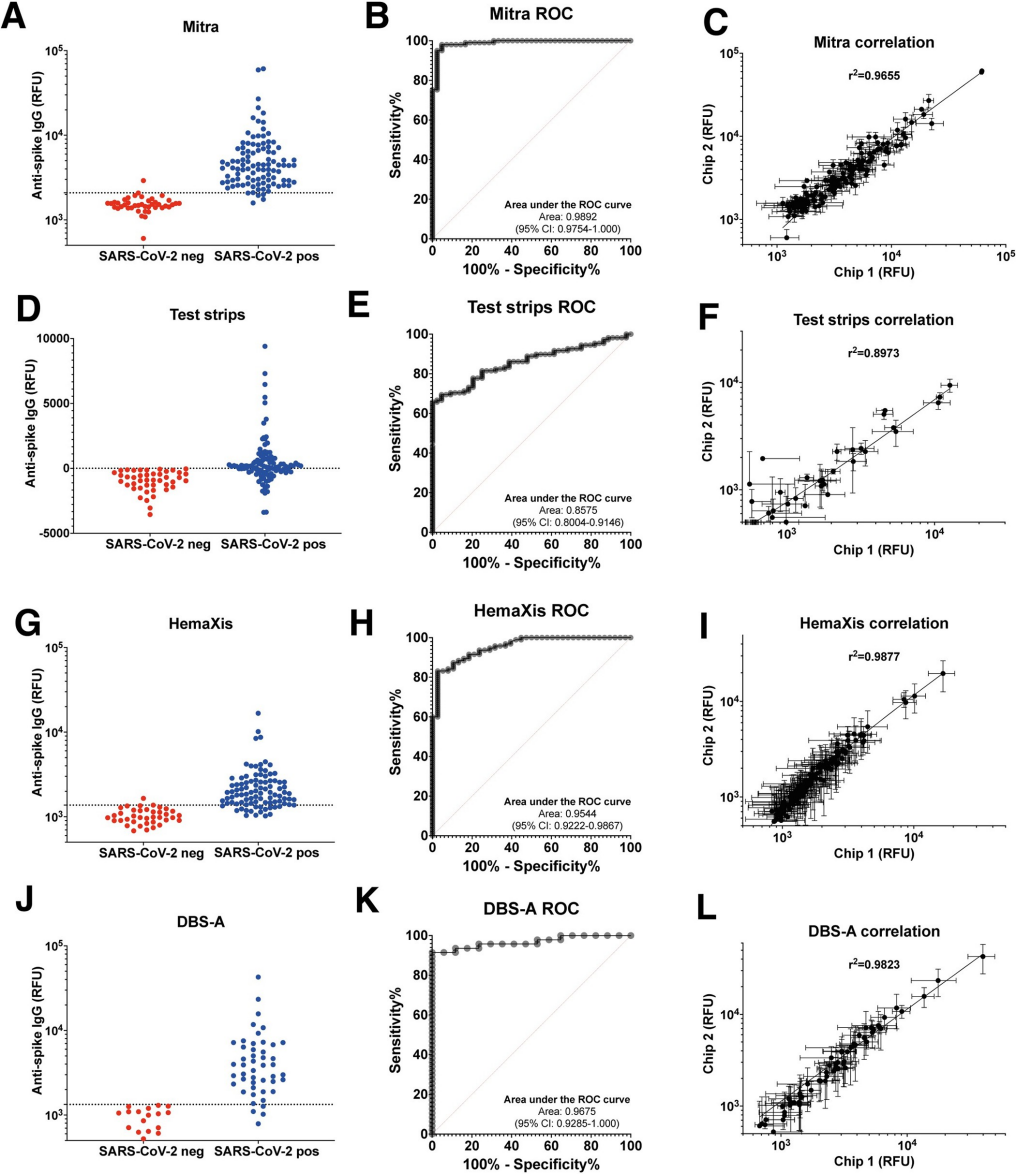
Performance of microfluidic nano-immmunoassay (NIA) test using dried blood microsamples. Signal intensities with positivity threshold (dotted line), ROC curve, and chip-to-chip correlation for the microfluidic nano-immunoassay testing of dried blood samples collected on Mitra (A-C), (glucose) test strips (D-F), or HemaXis microsampling devices (G-I), and after automated extraction by flow-through desorption of dried blood spots (DBS-A) (J-L).

Combining Mitra capillary blood collection with NIA resulted in very good discrimination between SARS-CoV-2 positive and SARS-CoV-2 negative samples (Fig 3A). The AUROC is 0·9892 (95% CI: 0·9754–1·000) (Fig 3B) and we determined a threshold for positivity using the maximum likelihood (>2089 RFU), which returns a clinical sensitivity of 95·05% (95% CI: 88·93–97·87) and specificity of 97·62% (95% CI: 87·68–99·88). The Mitra device gave the highest clinical sensitivity among the different microsampling devices. There is a decrease in signal compared to serum samples (Fig 2D), which is likely due to a higher dilution factor after the sample extraction step. Nonetheless, we obtain a PPV of 93·01%, a NPV of 98·34% and a diagnostic accuracy of 96·98% considering a 25% prevalence (Table 1). The reproducibility between two measurements (N = 143) on separate days again showed a good correlation with an R2 = 0·9655 (Fig 3C). The percentage agreement with the Roche reference assay was 95·80% with a Kappa coefficient of 0·902 (95% CI: 0·825–0·978) (Table 1). This excellent agreement with the Roche serum analysis is promising as it takes into account all additional steps such as microsampling, dried shipping from one institution to another, manual extraction, freezing of the extract, and finally testing on NIA.

### Glucose test strips

Using widely available and low-cost glucose test strips could potentially offer an alternative to currently available commercial devices, but the ultra-low volume of blood collected by glucose test strips is generally not sufficient for analysis with existing immunoassays. In our previous study, we showed as a proof-of- concept that it was technically feasible to measure small volume samples of venous blood sampled by the repurposed glucose test strips [22]. Here, combining capillary blood collection on glucose test strips with NIA showed modest discrimination between SARS-CoV-2 positive and SARS-CoV-2 negative samples (Fig 3D). Indeed, the background-subtracted signal is noticeably lower compared to serum at 1:8 dilution or using the other microsampling devices, with a significant number of samples displaying higher background signal in the microfluidic chamber outside of the MITOMI assay region, resulting in negative background-subtracted signals. The AUROC is 0·8575 (95% CI: 0·8004–0·9146) (Fig 3E) and the positivity threshold selected as the lowest non-negative background-subtracted signal (>8·167 RFU) gives a clinical sensitivity of 61·11% (95% CI: 51·69–69·77) and specificity of 100% (95% CI: 91·97–100·0). This represents a PPV of 100%, a NPV of 88·52% and a diagnostic accuracy of 90·28% (Table 1). The higher dilution factor when extracting ultra-low sample volume of ∼0·6 μL in 30 μL buffer leads to a decrease in signal which negatively impacts sensitivity. While the decrease in signal would still allow identification of subjects with sufficiently high antibody levels, it might be more challenging in populations in which subject antibody levels have decreased due to a long interval since previous infection as we find here. The reproducibility between two separate measurements (N = 152) showed a correlation with an R2 = 0·8973 (Fig 3F), and the percentage agreement with the Roche assay was lower compared to Mitra at 72·55% and a Kappa coefficient of 0·478 (95% CI: 0·364–0·593) (Table 1).

### HemaXis device

Combining capillary blood collection on Hemaxis device with NIA showed good discrimination between SARS-CoV-2 positive and SARS-CoV-2 negative samples (Fig 3G). The AUROC is 0·9544 (95% CI: 0·9222–0·9867) (Fig 3H) and the threshold for positivity using the maximum likelihood (>1372 RFU) gives a clinical sensitivity of 83·16% (95% CI: 74·38–89·36) and specificity of 97·37% (95% CI: 86·51–99·87). We note a decrease in signal from samples collected on HemaXis compared to the samples collected on the Mitra device, even though the sampled (10 μL) and extracted volumes (200 μL) were identical. One possible explanation is that sample extraction is generally less efficient due to the different materials used, or that extraction is slower for HemaXis and would require more than the 3h extraction step used in this study. Indeed, we didn’t observe any difference in our previous study where samples were extracted overnight at 4°C [22]. At a prevalence of 25%, these results represent a PPV of 91·33%, a NPV of 94·55% and a diagnostic accuracy of 93·82% (Table 1). The reproducibility between two measurements (N = 133) on different days showed a good correlation with an R2 = 0·9877 (Fig 3I). The percentage agreement with the Roche assay was 87·22% with a Kappa coefficient of 0·720 (95% CI: 0·600–0·840) (Table 1).

### HemaXis device and automated flow-through desorption

As manual punching of dried blood spots from filter paper cards requires considerable operator time, we also tested the use of an automated dried blood spots (DBS-A) extraction method [26]. We observed that using flow-through desorption with a fully automated instrument (DBS-A, Gesrtel AG) improved the discrimination between SARS-CoV-2 positive and SARS-CoV-2 negative samples collected on filter paper cards with the Hemaxis device (Fig 3J). The AUROC was 0·9675 (95% CI: 0·9285–1·000) (Fig 3K) and the threshold for positivity using the maximum likelihood (>1333 RFU) returned a clinical sensitivity of 91·49% (95% CI: 80·07–96·64) and specificity of 100% (95% CI: 81·57–100). When comparing only with the subset of corresponding samples, manual extraction of HemaXis would present a clinical sensitivity of 87·50% (95% CI: 75·30–94·14) and specificity of 100% (95% CI: 81·57–100). While automated extraction still corresponds to a clinical sensitivity improvement and an increase in assay signal, it would be necessary to analyze a larger proportion of the samples for a more accurate comparison. Also, the confidence intervals for the sensitivity and specificity evaluation were relatively large due to the smaller number of samples analyzed. The higher clinical sensitivity and specificity using DBS-A extraction together with a prevalence of 25% corresponds to a PPV of 100%, a NPV of 97·24% and a diagnostic accuracy of 97·87%. Finally, the reproducibility between two separate measurements (N = 65) showed a good correlation with an R2 = 0·9823 (Fig 3L), and the percentage agreement with the Roche assay was 93·75% with a Kappa coefficient of 0·851 (95% CI: 0·711–0·991) (Table 1).

### Comparison between serum and dried blood samples

Previous findings have shown that anti-SARS-CoV-2 antibodies are reliably eluted from whole blood loaded on Mitra microsamplers and present equivalent signal compared to serum samples after testing on ELISA [16]. Also, NIA is capable of providing information on antibody concentration in serum using a single measurement at 1:8 dilution [22]. Here, we investigate the correspondence between signals obtained using microsampling and matched serum samples obtained by venipuncture on the same day.

**Fig 4:**
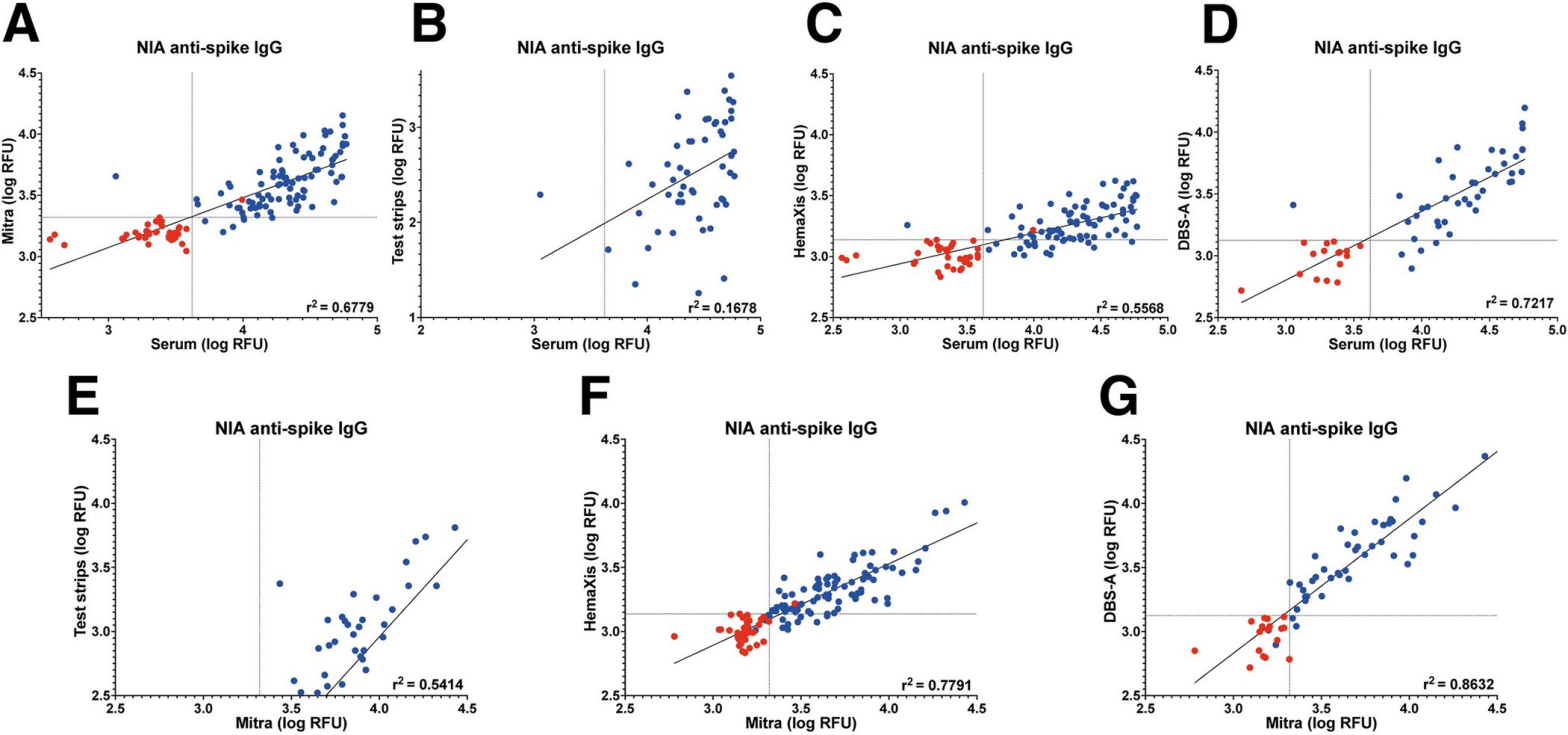
NIA serum and microsampling comparison. Comparison of serum at 1:8 dilution and samples obtained by microsampling tested on NIA. Samples from SARS-CoV-2 negative and SARS-CoV-2 positive participants are labeled red and blue, respectively. Comparison of serum samples with A) Mitra, B) (glucose) test strips, C) HemaXis, and D) HemaXis with automated extraction (DBS-A). Serum samples with signal above 59000 RFU were removed from the plot and linear regression. Comparison of Mitra with E) glucose test strips, F) HemaXis, and G) HemaXis with automated extraction (DBS-A). In panels B) and F), samples with negative signals were excluded. The samples from SARS-CoV-2 negative participants were not included in the linear regression.

We observed a modest correlation between serum samples and the dried blood microsample eluates tested on NIA with an R2 = 0·6779 for Mitra, R2 = 0·5568 for HemaXis, and R2 = 0·7217 for HemaXis after DBS-A extraction (Fig 4A, 4C, and 4D). On the other hand, using test strips for capillary microsampling leads to an absence of correlation with serum samples with an R2 = 0·1678 (Fig 4B). Capillary blood sampling of a defined micro-volume using Mitra or HemaXis, together with complete microsample extraction helps in avoiding the hematocrit bias associated with partial dried blood spot processing [27]. However, variation in hematocrit among individuals [28] could still contribute to some variation in the antibody concentration in capillary microsamples and explain the modest correlation observed. We therefore assessed the correlation between the samples obtained on different microsampling devices. While the correlation remains low when comparing samples obtained using Mitra and glucose test strips with an R2 = 0·5414 (Fig 4E), we see a good correlation between samples obtained using Mitra and HemaXis with an R2 = 0·7791 (Fig 4F), and an R2 = 0·8632 after DBS-A extraction (Fig 4G). This observation suggests that hematocrit levels or factors influencing intra-individual differences between serum and capillary blood antibody levels could be in part responsible for the observed variation in signal on NIA.

## Discussion

This study presents an extensive evaluation of micro-volume capillary blood sampling devices with analysis on a recently developed high-throughput microfluidic nano-immunoassay platform (NIA). Validation with serum samples confirmed the excellent performance of NIA. Of particular note is that samples in this study were obtained from participants with a long interval of 11 months between infection and blood sampling, which leads to decreases in antibody titers and therefore requires high-performance assay to return accurate results [29]. Moreover, the high performance of NIA on late time-point serum samples was obtained by measurements performed without any signal amplification, using only a few nanoliters of sample per assay, minute reagent consumption, and a throughput of 1024 assay points for each run on the microfluidic nano- immunoassay. While the clinical sensitivity of 98·33% in NIA was lower than the Elecsys anti-SARS-CoV- 2 S total Ig reference assay, it was higher than the commercial anti-SARS-CoV-2 S1 IgG ELISA having only 51·40% clinical sensitivity at 11 months post infection when using the cutoff recommended by the manufacturer. This observation is confirming previous results showing a decrease in clinical sensitivity for serum samples obtained from individuals infected many months prior to sample collection, and also points out that selection of the appropriate test or reevaluation of the cutoff might be necessary depending on the intended use of the assay, such as when performing serological surveys [26].

We also note that the antigen and format used in the different assays might lead to some differences in the seropositivity evaluation, as the Elecsys anti-SARS-CoV-2 S total Ig assay uses RBD antigen in a sandwich format detecting all antibody classes, while the anti-SARS-CoV-2 S1 IgG ELISA uses the S1 part of the spike protein immobilized on a plate and detects IgG antibodies only. On the other hand, NIA uses a full-length trimerized spike pulled down on the assay surface by an anti-His-tag antibody and detects IgG antibodies.

**Fig 5:**
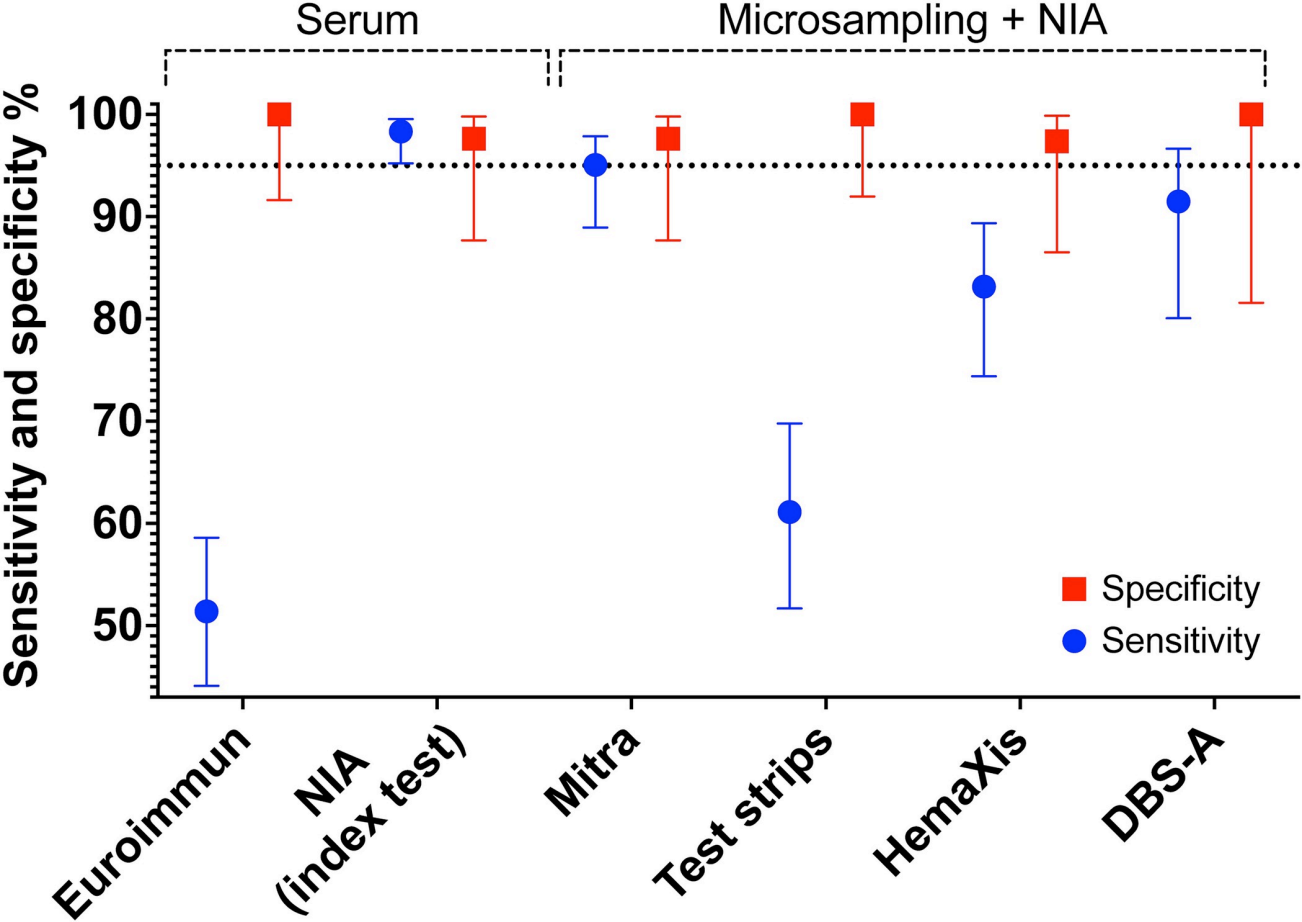
Sensitivity and specificity comparison. Sensitivity and specificity with 95% confidence interval are shown. Euroimmun ELISA and NIA index test were performed on serum obtained by venipuncture. Mitra, (glucose) test strips, and HemaXis correspond to the device used for capillary blood collection after fingerprick, and DBS-A to automated extraction of samples obtained on HemaXis. Capillary blood samples were dried, shipped, and extracted before testing on the NIA assay. A dotted line at 95% sensitivity or specificity is shown for visual aid.

Following validation using serum samples, evaluation of the combined use of NIA with samples obtained by capillary blood microsampling demonstrated its suitability for serological surveys (Fig 5). First, testing of capillary blood obtained by fingerprick using a Mitra microsampling device and analyzed with NIA offers performance characteristics comparable to tests performed in the laboratory using serum obtained by venipuncture, with a clinical sensitivity of 95·05% in participants 11 months post infection. The performance is better than a lateral flow immunoassay (LFIA) evaluated [30] and selected for a large-scale seroprevalence study [31], or other more recently developed LFIAs [32], which shows the utility of Mitra microsampling combined with NIA in conducting decentralized serological surveys.

Using glucose test strips, the clinical sensitivity is lower than using purpose built and professionally designed microsampling devices. However, with their low cost and wide availability, repurposed glucose test strips could play a role when conducting large serosurveillance studies, provided improvements can be made in the sampling or extraction parameters to allow sufficient detection of antibody signal in such challenging late time-point samples. On the other hand, the use of glucose test strips for early time-point samples is likely already of sufficient quality based on our previous results [22], although this remains to be assessed in a suitable cohort.

Collection of capillary blood on HemaXis and testing on NIA affords relatively good sensitivity and specificity when analyzed on NIA. Although the HemaXis device showed lower sampling success rates compared to Mitra or glucose test strips, the success in sampling is still relatively high at 86,2%. An advantage of using HemaXis compared to Mitra device is the wide use of filter paper for sample collection and the associated methods for processing the dried blood samples, such as automated punchers which are commonly used in neonatal screening programs [33]. To benefit from HemaXis volumetric sampling and to avoid hematocrit bias, it is however preferable to extract the entire dried blood spot. While manual punching and extraction offered lower sensitivity when compared to results obtained with Mitra, flow-through desorption of the entire dried blood spot using a fully automated dried blood spot extraction (DBS-A) improved the clinical performance. With barcode recognition capability and robotic processing, an efficient workflow can be envisioned in combination with the high-throughput of NIA.

We recognize different limitations in our study and acknowledge the following explicitly. We relied for the SARS-CoV-2 positive group on participants already involved in a longitudinal evaluation of anti-SARS-CoV-2 antibody levels and all participants had a positive result on serology at the baseline visit. While the serological status at the baseline did not constitute a selection criterium for the SARS-CoV-2 positive group, this can lead to an overestimation of the sensitivity as the SARS-CoV-2 positive group does not include potential patients with previous infection who did not develop an antibody response [25]. The participants were all presenting mild to moderate symptoms and no asymptomatic patients, nor patients with severe or critical illness requiring further care were included. The participants in the SARS-CoV-2 negative group formed a convenience sample which might not be fully representative of the general population, and 5 participants from the SARS-CoV-2 negative group had to be excluded from the study due to positive signal on the Roche reference assay. The specificity evaluation did not address some common interference in immunoassays such as for patients with autoimmunity or chronic inflammation and could be investigated further in a study including such patients. Moreover, we did not test cross-reactivity to other coronaviruses or other respiratory viruses which is another potential cause of false positives. Finally, the number of participants was not high, and resulted in relatively broad confidence intervals for the specificity evaluation (Fig 5), but also for the sensitivity evaluation when the observed sensitivity was lower than expected from previous results [22].

In conclusion, we evaluated in this study micro-volume capillary dried blood sampling methods combined with a high-throughput microfluidic nano-immunoassay (NIA) [22] and showed its potential for use in conducting serological surveys for COVID-19. In a cohort with SARS-CoV-2 positive participants 11 months post infection, the high clinical sensitivity and specificity of NIA was demonstrated when testing serum samples as well as dried blood microsamples obtained by fingerprick, which should facilitate its use in large-scale studies using home-based sampling. The simplified sample collection will also be useful when testing vulnerable populations where blood sampling by venipuncture is difficult to implement, such as in the elderly [34] or pediatric populations [35, 36]. Finally, the open design of NIA allows for the rapid modification of the assay allowing to perform serological surveys and gather information on present and future SARS-CoV-2 variants, other circulating pathogens of public health importance, as well as emerging infectious diseases.

## Supporting information

S1 Checklist STARD checklist. (XLSX)

S2 Checklist MIQE checklist. (DOCX)

S1 File Supplementary methods with further description of the methods used to perform the study. (DOCX)

S1 Table This containing the cross-tabulated assay results for every participant. (XLSX)

S1 Fig The microsampling kit. (TIF)
