# Supplementary material for: Clinical sensitivity and specificity of a high-throughput microfluidic nano-immunoassay combined with capillary blood microsampling for the identification of anti-SARS-CoV-2 Spike IgG serostatus
Source: PLoS One. 2023 Mar 23;18(3):e0283149. doi: 10.1371/journal.pone.0283149 (PMC10035827; doi:10.1371/journal.pone.0283149)
Supplement: S2 Checklist — (DOCX) [file pone.0283149.s002.docx]

**MIQE Checklist**

| \| **Category** \| **Item to be described/detailed** \| **Page No.** \| **Author Comments** \| \| --- \| --- \| --- \| --- \| \| **SAMPLE** \| Type (blood, etc.) \|  \| Blood \| \|  \| Method of dissection/procurement \|  \| Microsampling (Mitra, Hemaxis, glucose test strips) and venipuncture \| \|  \| Processing procedure \|  \| Venipuncture blood processed into serum  Microsampling capillary blood dried and extracted in buffer \| \|  \| If frozen, how and how quickly? \|  \| Serum Placed in -20°C \| \|  \| If fixed, with what and how quickly? \|  \|  \| \|  \| Storage conditions and duration \|  \| -20°C for serum  Room temperature for dried blood specimens \| \| **EXTRACTION** \| Method or instrument \|  \| Thermomixer (37°C, 3h, 300 rpm) \| \|  \| Reagents/kits/modifications \|  \|  \| \|  \| DNAse or RNAse treatment \|  \|  \| \|  \| Evidence for lack of contamination (DNA or RNA) \|  \|  \| \|  \| Nucleic acid quantification \|  \|  \| \|  \| RNA integrity \|  \|  \| \| **REVERSE TRANSCRIPTION** \| Complete reaction conditions, including all components and their concentrations \|  \|  \| \|  \| RNA amount and reaction volume \|  \|  \| \|  \| Priming oligo sequence(s) \|  \|  \| \|  \| Cqs with and without reverse transcriptase \|  \|  \| \| **qPCR TARGET** \| HUGO gene abbreviation \|  \|  \| \|  \| Sequence accession number \|  \|  \| \|  \| Amplicon length \|  \|  \| \|  \| *In silico* specificity (BLAST) \|  \|  \| \|  \| Location by exon/intron \|  \|  \| \|  \| Identify the splice variants amplified \|  \|  \| \|  \| All primer/probe sequences \|  \|  \| \|  \| Location and identity of any oligonucleotide modifications \|  \|  \| \| **qPCR PROTOCOL** \| Complete reaction conditions, including all components and their concentrations \|  \|  \| \|  \| cDNA/DNA amount and reaction volume \|  \|  \| \|  \| Instrument identification and complete thermocycling parameters \|  \|  \| \| **qPCR VALIDATION** \| Evidence for PCR specificity (gels, sequencing, or melting curves) \|  \|  \| \|  \| Template inhibition data (template titrations) \|  \|  \| \|  \| For SYBR Green I reactions, the Cq of the no template control \|  \|  \| \|  \| Calibration curves with slope and intercept \|  \|  \| \|  \| PCR efficiency from the slope \|  \|  \| \|  \| r^2^ of the calibration curve \|  \|  \| \|  \| Evidence for the linear dynamic range \|  \|  \| \|  \| Evidence for the limit of detection \|  \|  \| \|  \| For multiplexed assays, the efficiency and limit of detection of each assay \|  \|  \| \| **DATA ANALYSIS** \| qPCR analysis method/software \|  \|  \| \|  \| Method of Cq determination \|  \|  \| \|  \| Results of no template controls \|  \|  \| \|  \| Justification of number and choice of reference genes \|  \|  \| \|  \| Normalization method \|  \|  \| \|  \| Number and stage (reverse transcription or qPCR) of technical replicates \|  \|  \| \|  \| Intra-assay variation in terms of concentration, not Cq \|  \|  \| \|  \| Statistical methods/software \|  \|  \| |
| --- | --- | --- | --- | --- | --- | --- | --- | --- | --- | --- | --- | --- | --- | --- | --- | --- | --- | --- | --- | --- | --- | --- | --- | --- | --- | --- | --- | --- | --- | --- | --- | --- | --- | --- | --- | --- | --- | --- | --- | --- | --- | --- | --- | --- | --- | --- | --- | --- | --- | --- | --- | --- | --- | --- | --- | --- | --- | --- | --- | --- | --- | --- | --- | --- | --- | --- | --- | --- | --- | --- | --- | --- | --- | --- | --- | --- | --- | --- | --- | --- | --- | --- | --- | --- | --- | --- | --- | --- | --- | --- | --- | --- | --- | --- | --- | --- | --- | --- | --- | --- | --- | --- | --- | --- | --- | --- | --- | --- | --- | --- | --- | --- | --- | --- | --- | --- | --- | --- | --- | --- | --- | --- | --- | --- | --- | --- | --- | --- | --- | --- | --- | --- | --- | --- | --- | --- | --- | --- | --- | --- | --- | --- | --- | --- | --- | --- | --- | --- | --- | --- | --- | --- | --- | --- | --- | --- | --- | --- | --- | --- | --- | --- | --- | --- | --- | --- | --- | --- | --- | --- | --- | --- | --- | --- | --- | --- | --- | --- | --- | --- |
|  |
