# Supplementary material for: Clinical sensitivity and specificity of a high-throughput microfluidic nano-immunoassay combined with capillary blood microsampling for the identification of anti-SARS-CoV-2 Spike IgG serostatus
Source: PLoS One. 2023 Mar 23;18(3):e0283149. doi: 10.1371/journal.pone.0283149 (PMC10035827; doi:10.1371/journal.pone.0283149)
Supplement: S1 File — (DOCX) [file pone.0283149.s003.docx]

**Materials and methods**

**Participants and study design**

We did not perform a sample size calculation. We aimed at obtaining over 100 participants in the SARS- CoV-2 positive group and over 50 participants in the SARS-CoV-2 negative group based on participants availability. This would allow a lower limit of the 95% confidence interval above 85% in the sensitivity and specificity evaluation [1], assuming equivalent sensitivity and specificity as our previous results based on serum analysis [2]. Adult participants with a documented PCR-positive SARS-CoV-2 infection were eligible in the positive group, and participants without knowledge of previous SARS-CoV-2 infection were eligible in the negative group. The patients in the positive group were already identified and part of another study [3] and had a documented previous infection 6-12 months before the start of this study. Between February and March 2021, 200 participants were invited to participate in a follow-up serology using venous blood as part of ongoing studies and were solicited for their participation in this additional investigation using capillary blood microsampling. Participants in the negative group, which were recruited specifically for this study, consisted of a convenience sample of non-clinical employees of the University of Geneva or Geneva University Hospital. The corresponding study diagram in Fig 1 was created with the Lucidchart web application. This study was approved by the local ethics committee (CCER project numbers 2020- 00516 and 2020-02323) and registered (NCT04329546) prior to initiation.

**Sample collection and preparation**

*Serum samples*

Serum samples obtained from venipuncture were available for participants as part of the investigations of the persistence of anti-SARS-CoV-2 antibodies [3]. Aliquots were retrieved from the existing blood bank, heat inactivated at 56◦C and Triton X-100 was added to each sample to a concentration of 1%. The inactivated samples were shipped refrigerated by post from Geneva to Lausanne for analysis.

*Capillary blood microsampling*

Each capillary microsampling kit contained: general material for capillary blood sampling (3 lancets (BD Microtainer High Flow - 21 G 2·0 mm), pre injection cleansing swab (PDI,70% isopropyl alcohol), gauze, plasters, return sample bag with dessicant, return box), 1 Mitra sampling device in cartridge format with 2 x 10 μL sample collectors in a resealable sample bag containing dessicant (Neoteryx), 1 Hemaxis device with 4 x 10 μL sampling channels (DBS System SA), and 2 x 0·6 μL glucose test strip (Medisana MediTouch 2) placed in 2 mL tubes with pierced cap to allow sample drying (S1 Fig). Except for one participant who collected the samples at home, all samples were collected at the Geneva University Hospital with the supervision and help of research staff. After de-identification, samples were sent by post every week in a package containing samples from the previous week.

*Sample processing*

To minimize the risk of exposure to biohazardous agents, the samples were extracted after more than 4 days in a dried state. The samples were extracted in a buffer containing surfactants, namely phosphate buffered saline (PBS) containing 1% bovine serum albumin (BSA) and 0·5%Tween-20 (PBS-BT) [4]. The Mitra tips were separated from the stem and Hemaxis spots on paper cards were punched out with an 8 mm puncher and were each placed in a well of a 96-well plate and 200 μL PBS-BT was added to each well (10 μL sample = 20x dilution factor). The plates were incubated at 37◦C for 3h on a ThermoMixer C with 300 rpm agitation and a ThermoTop lid to prevent condensation (Eppendorf). For glucose test strips, the 0·6 μL samples were extracted in 30 μL PBS-BT (50x dilution factor) for the test strip to be sufficiently immersed in the extraction buffer at the bottom of the 2 mL tube and were incubated at 37◦C for 3h without agitation. All extracted samples were stored at -20◦C until analysis.

*Automated DBS extraction*

For the automated extraction of DBS on filter paper cards, we used a dried blood spot autosampler [5] (Gerstel SA). A 6mm clamp was mounted to extract samples by flow-through desorption using PBS-BT, and 2 x 150 μL were flowed at a speed of 700 μL/min through the spot allowing collection of 2 fractions. To improve spot desorption, the extraction buffer was briefly heated to 80◦C before flowing through the spot. The first fraction was discarded as it corresponded to air void volume or washing buffer contained in the tubing after the clamp. The second fraction was collected in a 96-well round-bottom microtiter plate (Nunc) corresponding to a volume of around 80 μL with the rest of the 150 μL remaining in the tubing and eliminated in subsequent washing steps. Between samples, a washing of all the tubing and lines was performed with 1000 μL of extraction buffer at a speed of 4000 μL/min.

**Microfluidic nano-immunoassay**

*Microfluidic chip fabrication*

For the microfluidic chip fabrication, we used the same protocol as described previously without modification [2].

*Microarray spotting*

The extracted samples were thawed on ice and 25 μL were transferred to a low-volume 384-well plate (Arrayit, MMP384). Each sample was deposited into 4 spots using a randomized spotting pattern, and two stamps per spot with an inking time of 50 ms and printing time of 1 ms. Other microarray spotting parameters were kept as described previously [2].

*Immunoassay reagents*

We used the same reagents as described in our previous study [2]. BSA-biotin (Thermo Fisher, 29130) and neutrAvidin (Thermo Fisher, 3100) were used for surface modification of the microfluidic device. We used biotinylated mouse anti-His antibodies (Qiagen Cat# 34440, RRID:AB 2714179) followed by full length His-tagged SARS-CoV-2 Spike produced at the EPFL protein facility. The prefusion ectodomain of SARS- CoV-2 spike glycoprotein (the construct was a generous gift from Prof. Jason McLellan, University of Texas, Austin [6]) was transiently transfected into suspension-adapted HEK293 cells (Thermo Fisher) with PEI MAX (Transfection grade linear polyethylenimine hydrochloride, Polysciences) in Excell293 medium. Incubation with agitation was performed at 37◦C and 4·5% CO2 for 5 days. The clarified supernatant was loaded onto Fastback Ni2+ Advance resin column (Protein Ark) eluted with 500 mM imidazole, pH 7·5 in PBS. For detecting human IgG, we used PE labeled goat anti-Human-IgG (Abcam Cat# ab131612, RRID:AB 11156857).

*Running on-chip immunoassays*

We used the same procedure described in a previous study when performing the microfluidic nano-immunoassay [2]. Briefly, the microfluidic assay allows the successive flowing of immunoassay reagents and resolubilization of
spotted sample which are individually assayed in a unit cell. The unit cells were imaged using an automated microscope stage. In addition to a fluorescent image, a brightfield image was acquired for each unit cell at the same coordinates for use in the image analysis.

*Image analysis*

We used FIJI and a custom macro to process the image stacks for the 1024 unit cells. Using the brightfield image, a circular region corresponding to the MITOMI valve area was detected using a Hough Circle Transform plugin (UCB Vision Sciences library). An ROI defined as the button area was obtained after 30 steps of erosion of the MITOMI valve region. A second ROI defined as the background area was obtained after 30 dilation steps of the MITOMI valve region and subtracted by the button area ROI. The final signal for each unit cell was calculated as the median of the button area subtracted by the median of the background area. For each sample, the mean and standard deviation of 4 technical repeats was calculated.

**Selection of reference assay**

The Roche anti-SARS-CoV-2 S immunoassay was selected because of its reported high sensitivity and specificity by the manufacturer, and studies performed independently concluded in the high specifictity and sensitivity of 99.95% (95% confidence interval [CI]: 99.87–99.99; 7876/7880) and 97.92% (95% CI: 95.21–99.32; 235/240), respectively, for samples ≥14 days post-PCR [7]. It was also previously shown to achieve high sensitivity in subjects with long interval since time of infection [8]. The Euroimmun anti-SARS-CoV-2 S1 IgG ELISA was also selected for comparison as it was the first CE-marked serological assay targeting part of the SARS-CoV-2 spike antigen [9] and was extensively validated in the Geneva Center for Emerging Viral Diseases [10].

**Roche Elecsys anti-SARS-CoV-2 S RBD total Ig**

SARS-CoV-2 specific antibodies were determined using the quantitative Elecsys S RBD total Ig assay on a cobas e801 analyser (Roche Diagnostics, Rotkreuz, Switzerland) in the clinical laboratory of the University of Geneva Hospital. Results are reported as concentrations (U/mL) and positivity was determined by using the manufacturer’s cuf-off >0·8 U/mL.

**Euroimmun anti-S1 IgG**

Euroimmun anti-SARS-CoV-2 S1 IgG ELISA (Euroimmun AG, Lübeck, Germany # EI 2606-9601 G) was performed manually according to the manufacturer’s instructions. The reactivity of each sample was measured at an optical density of 450nm (OD450) and then divided by the OD450 of the calibrator provided with each ELISA kit to minimize inter-assay variation. The quantitative results obtained were then expressed in arbitrary units and interpreted as follows: OD ratio: <0·8 = negative; ≥0·8 and <1·1 = borderline; ≥1·1 = positive.

**Statistics**

Statistical analyses were performed using GraphPad Prism Version 9.3.1. Every sample was tested in four technical replicates and the mean was used in ROC analysis. The threshold for positivity was selected as the maximum likelihood ratio calculated and the corresponding sensitivity and specificity with 95% confidence interval were obtained. The receiver operating characteristic curve and area under the curve with 95% confidence interval was also calculated. To estimate the concordance with the Roche assay, Kappa coefficient calculation was done using the online tool available at https://www.graphpad.com/quickcalcs/kappa1/. For the reproducibility between two microfluidic experiments, the mean and standard deviation of four technical replicates on each experiment was used, and a simple linear regression was performed using GraphPad Prism. For the comparison between serum and the different microsampling methods, a log transform of the mean was calculated and a simple linear regression was performed using GraphPad Prism.

**Ethical statement**

This study was approved by the local ethics committee (CCER project numbers 2020-00516 and 2020- 02323) and registered (NCT04329546) prior to initiation.

**Role of the funding sources**

The funding sources did not play any role in the study design, in the collection, analysis and interpretation of the data, in the writing of the report, or in the decision to submit the paper for publication.

**References**

1. Flahault A, Cadilhac M, Thomas G. Sample size calculation should be performed for design accuracy in diagnostic test studies. Clin Epidemiol 2005;58:859–862.

2. Swank Z, Michielin G, Yip HM, Cohen P, Andrey DO, Vuilleumier N, et al. A high-throughput microfluidic nanoimmunoassay for detecting anti–SARS-CoV-2 antibodies in serum or ultralow-volume blood samples. Proc Natl Acad Sci U S A 2021;118:e2025289118.

3. L'Huillier AG, Meyer B, Andrey DO, Arm-Vernez I, Baggio S, Didierlaurent A, et al. Antibody persistence in the first 6 months following SARS-CoV-2 infection among hospital workers: a prospective longitudinal study. Clin Microbiol Infect 2021; 27:784e1–784e8.

4. Klumpp-Thomas C, Kalish H, Drew M, Hunsberger S, Snead K, Fay MP, et al. Standardization of ELISA protocols for serosurveys of the SARS-CoV-2 pandemic using clinical and at-home blood sampling. Nat Commun 2020.

5. Knoop A, Geyer H, Lerch O, Rubio A, Schrader Y, Thevis M. Detection of anti-SARS-CoV-2 antibodies in dried blood spots utilizing manual or automated spot extraction and electrochemiluminescence immunoassay (ECLIA). Anal Sci Adv 2021;2:440-446.

6. Wrapp, D., Wang, N., Corbett, K. S., Goldsmith, J. A., Hsieh, C. L., Abiona, O., et al. Cryo-EM structure of the 2019-nCoV spike in the prefusion conformation. Science 2020;367(6483):1260–1263.

7. Riester, E., Findeisen, P., Hegel, J. K., Kabesch, M., Ambrosch, A., Rank, et al. Performance evaluation of the Roche Elecsys Anti-SARS-CoV-2 S immunoassay. Journal of virological methods 2021;297:114271.

8. Perez-Saez J, Zaballa M, Yerly S, Andrey DO, Meyer B, Eckerle I, et al. Persistence of anti-SARS-CoV-2 antibodies: immunoassay heterogeneity and implications for serosurveillance. Clin Microbiol Infect 2021;27:1695e7–1695e12.

9. Otter AD, Brown A, D’Arcangelo S, Bailey D, Semper A, Hewson J, et al. Implementation and Extended Evaluation of the Euroimmun Anti-SARS-CoV-2 IgG Assay and Its Contribution to the United Kingdom’s COVID-19 Public Health Response. Microbiol Spectr 2022;10:e0228921.

10. Meyer B, Torriani G, Yerly S, Mazza L, Calame A, Arm-Vernez I, et al. Validation of a commercially available SARS-CoV-2 serological immunoassay. Clin Microbiol Infect 2020;26:1386–1394.
