# Supplementary figures and images for: Clinical sensitivity and specificity of a high-throughput microfluidic nano-immunoassay combined with capillary blood microsampling for the identification of anti-SARS-CoV-2 Spike IgG serostatus
Source: PLoS One. 2023 Mar 23;18(3):e0283149. doi: 10.1371/journal.pone.0283149 (PMC10035827; doi:10.1371/journal.pone.0283149)

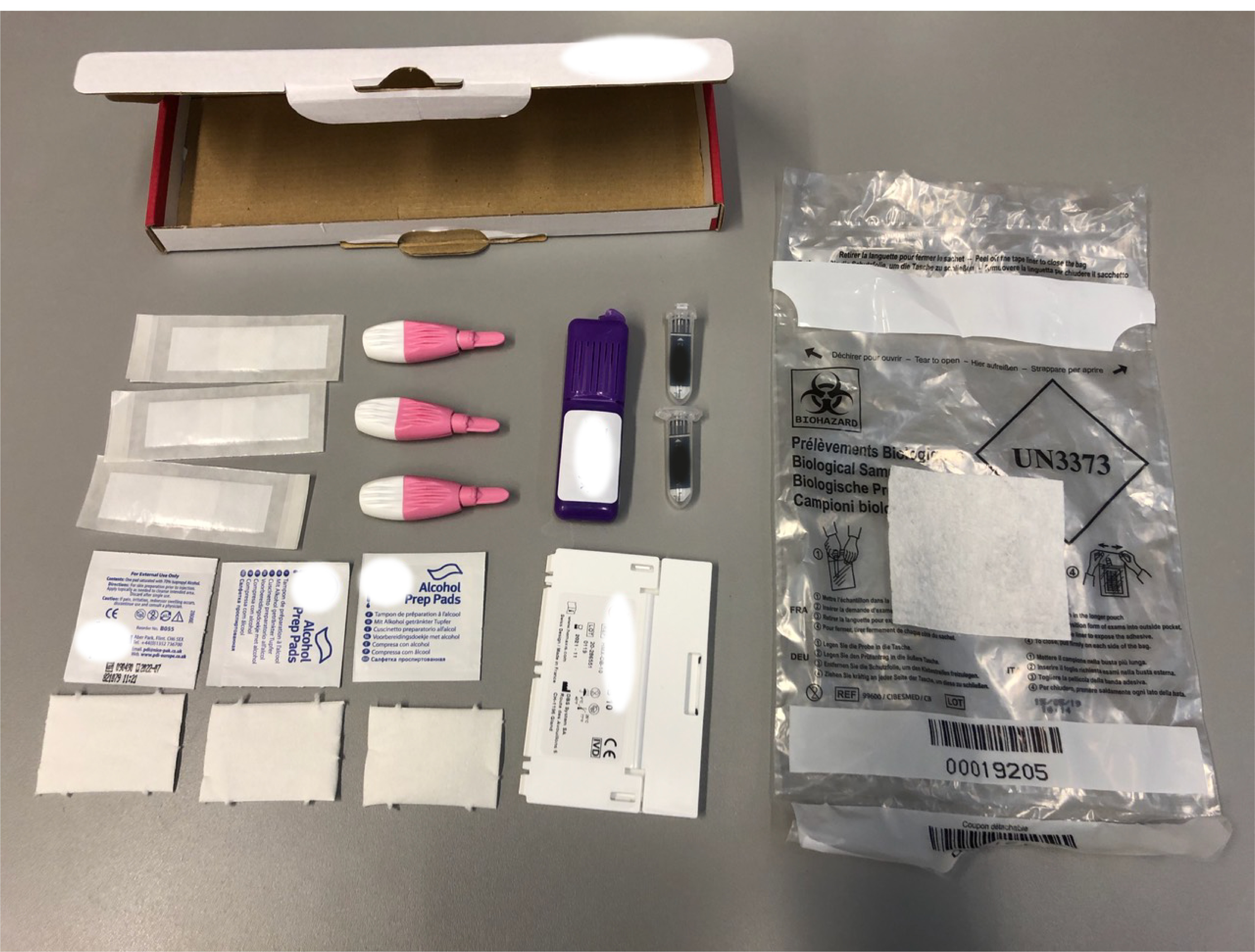

Supplement: S1 Fig — (TIF) [file pone.0283149.s005.tif]
